# Epidemiological changes and molecular characteristics of *Brucella* strains in Ningxia, China

**DOI:** 10.3389/fmicb.2024.1320845

**Published:** 2024-01-19

**Authors:** Guangtian Liu, Xueping Ma, Ruiqing Zhang, Jufen Lü, Pan Zhou, Bofei Liu, Tao Liu, Hui Ren, Zhiguo Liu, Zhenjun Li, Xuefeng Jiang

**Affiliations:** ^1^The Fourth People’s Hospital of Ningxia Hui Autonomous Region, Yinchuan, China; ^2^Ningxia Hui Autonomous Region Center for Disease Control and Prevention, Yinchuan, China; ^3^The College of Public Health, Ningxia Medical University, Yinchuan, China; ^4^National Key Laboratory of Intelligent Tracking and Forecasting for Infectious Diseases, National Institute for Communicable Disease Control and Prevention, Chinese Center for Disease Control and Prevention, Beijing, China

**Keywords:** human brucellosis, epidemiology, bacteriology, MLVA, molecular

## Abstract

**Objective:**

Human brucellosis causes serious public health concerns in Ningxia, China.

**Methods:**

This study employed epidemiological, bacteriological, and multiple-locus variable-number tandem repeat analysis (MLVA) methods to conduct an epidemiological investigation, which is necessary for devising tailored control strategies.

**Results:**

Between 1958 and 2022, 29,892 cases were reported, with an average annual number of cases and incidence of 467 and 7.1/100,000, respectively. The epidemic situation gradually worsened, with cases escalating from 26 cases in 2005 to 6,292 in 2022, with the incidence rate rising from 0.441 in 2005 to 86.83 in 2022. Geographically, the disease spread from a single affected county in 2004 to encompass all 22 counties in 2022. Yanchi County had the highest incidence, followed by the Hongsibao and Tongxin counties. These data suggest that *Brucella* infection has become a rampant regional concern in human brucellosis. Between 1958 and 2019, a total of 230 *Brucella* strains were identified across four studied hosts. These strains comprised four species with 12 biovars, including *B. melitensis* bv. 1, bv. 2, bv. 3, *B. abortus* bv. 1, bv. 3, bv. 4, bv. 5, bv. 6, bv. 7, *B. suis* bv. 1 and bv. 3, and *B. canis*. These data highlight the high species/biovars and host diversity of the *Brucella* population, posing a substantial challenge to brucellosis surveillance. There was an apparent transition from multiple species/biovars historically to the current dominance of a single species, *B. melitensis*, emphasizing the requirement for strengthening surveillance of *B. melitensis*. Genotypes 42 and 116, constituting 96.2% of the total number of genotypes, predominated in panel 1 and *MLVA-11*, indicating that all strains belong to the East Mediterranean lineage. *MLVA* cluster analysis revealed persistent transmission of dominant circulating genotypes, presenting an epidemic pattern characterized primarily by epidemiologically related cases with a few sporadic cases. Strains in this study exhibited high genetic homogeneity with strains from the Northwest, and those from Kazakhstan and Mongolia.

**Conclusion:**

The epidemic situation of human brucellosis has gradually worsened; the rampant epidemic of the disease has become a regional concern. The present study highlights that implementing the of targeted surveillance and intervention strategies is urge.

## Introduction

Human brucellosis, caused by *Brucella* spp., is a global public health concern (Pappas et al., 2006; Di Bonaventura et al., 2021). This disease exerts negative effects on human health and the animal farming industry. *Brucella* strains can result in severe clinical manifestations in the population, including fever, sweat, and fatigue, along with substantial economic losses in animal farming due to factors such as abortion and decreased milk productivity (Bagheri Nejad et al., 2020; Jamil et al., 2021). Although some developed countries have nearly eliminated this disease, the majority of low-income countries remain vulnerable to this zoonotic threat, including Africa, Latin America, Asia, and China (Gwida et al., 2010; Zhu et al., 2020). Since the 2000s, the incidence of human brucellosis has been continuously increasing and is distributed across all mainland regions in China, including the Ningxia Hui Autonomous Region (Ningxia) (Lai et al., 2017). Ningxia has a history of being an endemic area for brucellosis. The first survey of human brucellosis was conducted from 1958 to 1962, involving the sampling of 18,698 people, among whom 3,185 tested positive, resulting in a positivity rate of 17.03%. At that time, immunization had not yet been widely promoted, leading to the infection rate gradually increasing. Subsequently, a second brucellosis survey conducted from 1974 to 1980 revealed a positivity rate of 4.34% (68,609/1580,082). Following the implementation of mass ruminant immunization, the human brucellosis epidemic has been relatively stable, with 76.19% of the counties in the region reaching the national standard for stable brucellosis control (Deqiu et al., 2002).

Although vaccination and test-slaughter program animals, and surveillance and intervention plan in human population were exerted continuously. Since 2008, human brucellosis has steadily increased, with the current incidence surpassing historical levels. Conducting timely epidemiological analysis of resurging brucellosis areas is vital to enhance disease surveillance and improve intervention and treatment outcomes (Franco et al., 2007; Yagupsky et al., 2019; Matle et al., 2021). Additionally, pathogen surveillance is necessary for the formulation of targeted control measures and to provide a source for molecular epidemiological investigations (Laine et al., 2022). Therefore, the aim of this study was to illustrate the epidemiology of human brucellosis and the molecular association of *Brucella* strains in Ningxia to provide valuable insights for the development of targeted measures to halt its spread. To achieve this, we employed epidemiological analysis, pathogen surveillance, and molecular epidemiological approaches, including multiple-locus variable-number tandem repeat analysis (MLVA), which has become a routine epidemiological tool for distinguishing isolates from specific geographic origins and conducting trace-back analyses (Kiliç et al., 2011; De Massis et al., 2015).

## Methods

### Epidemiological data collection and processing, spatial cluster analysis

Data including the number of human brucellosis cases and incidence rates in Ningxia from 2003 to 2022 were obtained from the China Information System for Disease Control and Prevention, while epidemic data from 1958 to 2002 were acquired from the Annals of Health in China. Epidemic indices, such as cases and incidence rates, were utilized to depict brucellosis epidemic characteristics. The distribution maps of the annual incidence rate of human brucellosis from 1958 to 2022 were visualized using ArcGIS version 10.7 software (Esri; Redlands, California, United States), based on city boundaries. Subsequently, global spatial autocorrelation analysis was performed using the spatial autocorrelation tool in ArcGIS software (Version 10.6, Environmental Systems Research Institute, Redlands, United States). The global Moran’s *I* value served as a measure, where Moran’s *I* = 0 indicates that the regionalized variables are randomly distributed in space, Moran’s *I* > 0 suggests a positive correlation, and Moran’s *I* < 0 implies a negative correlation. Local autocorrelation is present only when |*z*| ≥ 1.96 and *p* ≤ 0.05 (*α* = 0.05). Local Moran’s *I* significance maps and cluster maps were utilized to illustrate the four different types of local correlation and the significance of the corresponding Moran’s *I* index. High–high regions denote geographical areas with a high incidence of brucellosis, encircled by other areas also exhibiting high incidences. Conversely, low–low regions represent geographical locations with low incidences of brucellosis, surrounded by areas with similarly low incidences. High–high and low–low regions signify clusters of geographic regions with analogous brucellosis incidence values, whereas high–low and low–high regions indicate spatial outliers (Luc, 1995).

### Sample collection, bacteriological isolation, and bio-typing of *Brucella* strains

The sampling data of strains isolated from the 1950s–1980s historically collected, sampling information is missing. After 2009, strains were isolated from human blood samples, 570 blood samples of patients were collected, and *Brucella* strains were isolated using a bacteriological approach (Di Bonaventura et al., 2021). Briefly, 5–10 mL of fresh blood was sampled and injected into a biphasic culture flask within a biosafety cabinet, followed by incubation at 37°C for a minimum of 30 continuous days. The flask was gently shaken after observation every other day. The Rose Bengal plate test and serum agglutination test were used for brucellosis screening and diagnosis (Araj, 2010). The suspected clones were further incubated, and bio-typing was identified using phenotypic methods previously described (Yagupsky et al., 2019), including CO_2_ requirement for growth, H_2_S production, growth inhibition by basic fuchsin and thionin, agglutination with monospecific antisera (A and M), and the phage lysis test.

### DNA extraction and MLVA genotyping of *Brucella* strains in this study

DNA from 105 (out of 230) available strains was prepared according to the manufacturer’s protocol (Qiagen, Heidelberg, Germany). All examined strains were confirmed to be *B. melitensis* through the detection of a 731 bp band specific to this species by AMOS-PCR (Bricker and Halling, 1994). Subsequently, these 105 *B. melitensis* strains, comprising 48 strains from 2019, 21 from 2013, 10 from 2012, 3 from 2011, 4 from 2010, 6 from 2009, 12 from 1979, and 1 from 1973 (Supplementary Table S1), were subjected to the MLVA approach (Le Flèche et al., 2006; Liu et al., 2019) for cluster analysis. Briefly, the genotyping approach, as previously described (Li et al., 2020), involved using panel 1 (MLVA-8), MLVA-11, and MLVA-16 to characterize the strains.

Polymorphisms at each locus and panels were analyzed using the Hunter-Gaston Diversity index (Hunter, 1990). Phylogenetic analysis was performed using unweighted pair group method using arithmetic averages in Bionumerics version 5.1 (Applied Maths, St-Martens-Latem, Belgium). To further explore the molecular epidemiology of *B. melitensis* strains at national (*n* = 595) (Supplementary Table S2) from previous study (Zhu et al., 2020) and international scales (*n* = 750, Supplementary Table S3) (MLVA genotyping database V1.4.0, https://microbesgenotyping.i2bc.paris-saclay.fr/databases), and genetic relationships between the 124 isolates were evaluated using a minimum spanning tree (MST) constructed by PHYLOViZ 2.0 software (Nascimento et al., 2016).

## Results

### Epidemiological characteristics of human brucellosis from 1958 to 2022

**Figure 1:**
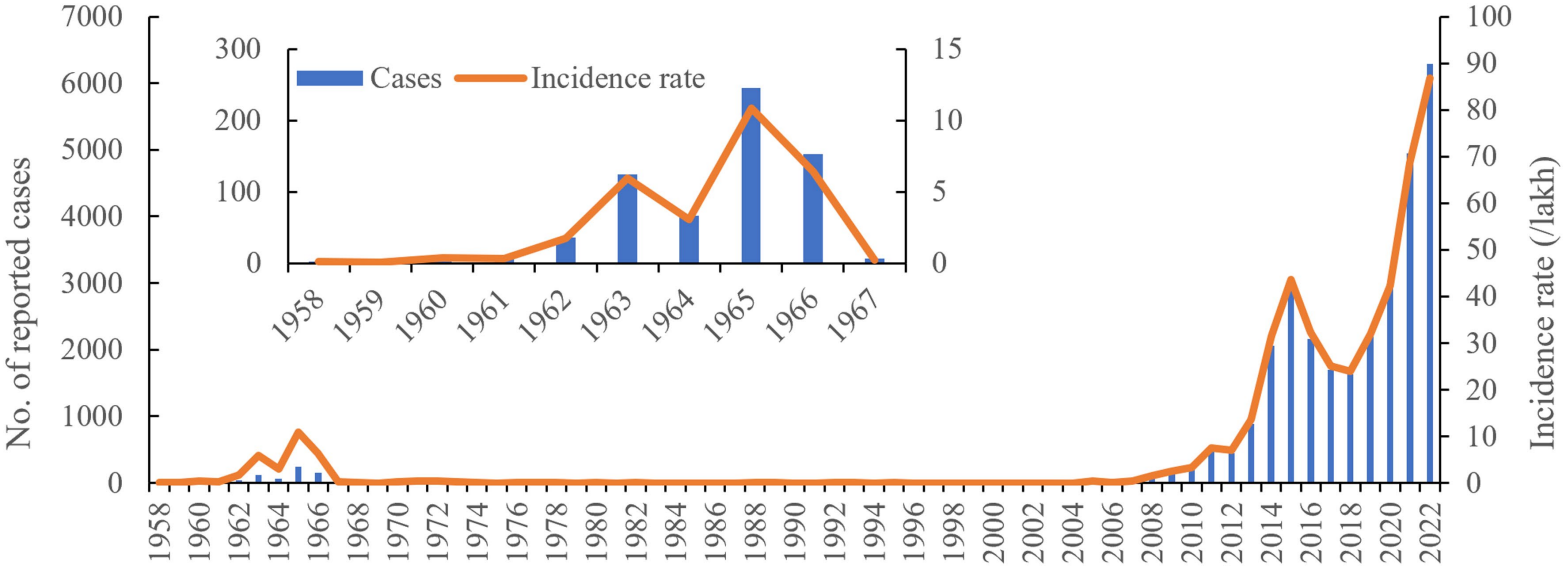
Human brucellosis epidemic profile between 1958 and 2022 in Ningxia, China.

A total of 29,892 cases were reported between 1958 and 2022, with an average annual number of cases and incidence rates of 467 and 7.1/100,000, respectively. Most cases (6,295) were reported in 2022, with an incidence rate of 86.83/100,000 (Figure 1). The epidemic phases of human brucellosis in Ningxia can be divided into three phases. The first phase, spanning from 1958 to 1967, was characterized as a low epidemic period, with an incidence rate increasing from 0.05/100,000 in 1959 to 10.87/100,000 in 1965. This was followed by a controlled phase from 1968 to 2004, with an incidence rate consistently below 0.45/100,000. Since 2005, it has entered a re-emerging epidemic phase. After 2004, the number of cases gradually increased annually, rising from 26 cases in 2005 to 6,292 in 2022. The incidence per 100,000 increased from 0.441 in 2005 to 86.83 in 2022.

### Geographic distribution and spatial autocorrelation analysis of human brucellosis

**Figure 2:**
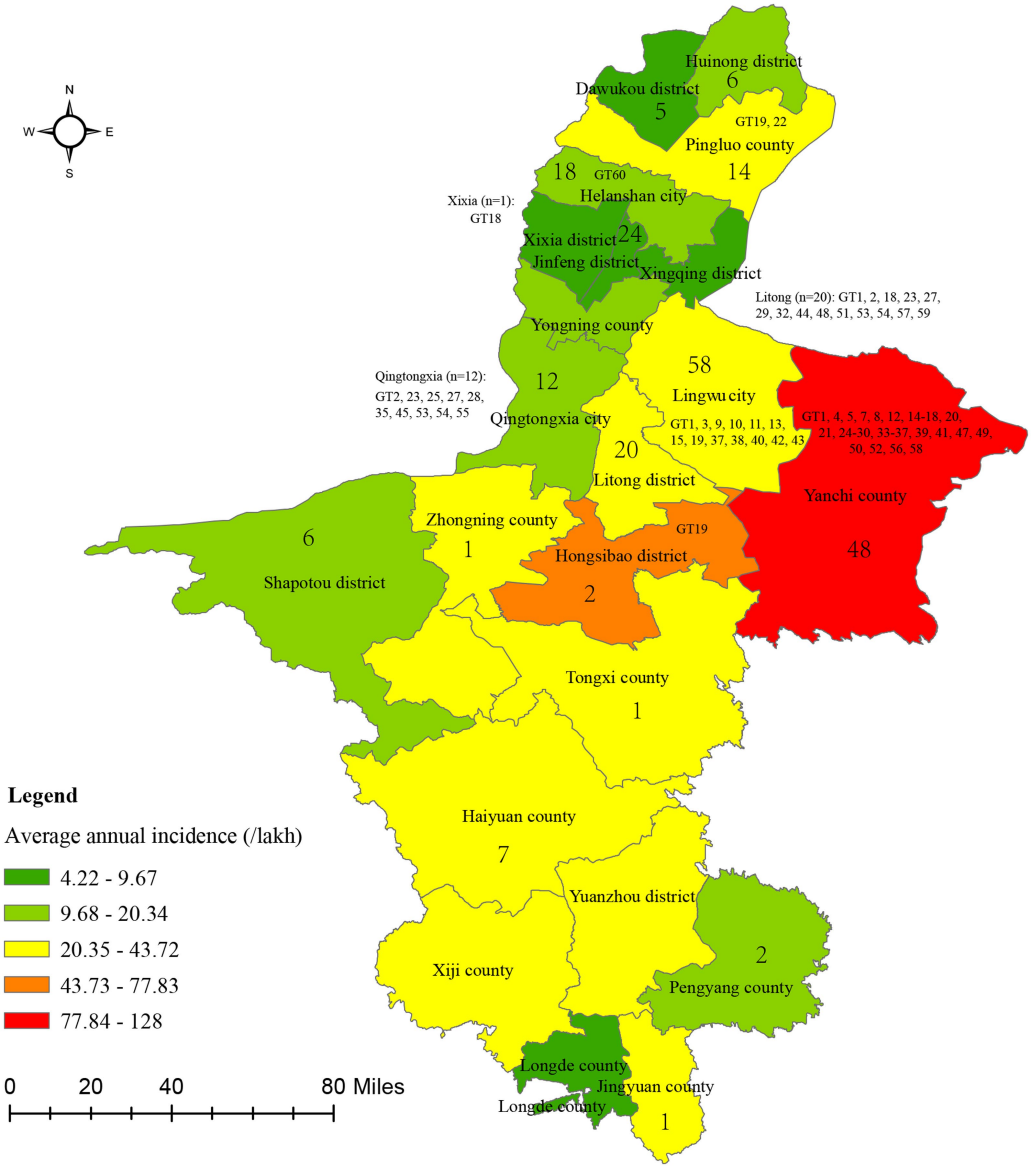
The average annual incidence of brucellosis across 22 counties in Ningxia from 2004 to 2022. Numbers in the figure showed the territory (*n* = 230) and MLVA-16 genotypes (*n* = 100) distribute profile of *Brucella* strains.

Geographically, the affected area continuously expanded from a single county in 2004 to encompass all 22 counties in 2022 (Figure 2). The cumulative number of cases across geographical regions ranged from 220 to 3,679, and the incidence rate ranged from 4.22/100,000 to 127.99/100,000 (Figure 2). Yanchi county reported the highest number of cases (3,679), followed by Tongxi county (2,702) and Hongsibao district (2,679), with average annual incidence rates (/100,000) of 127.99, 43.72, and 77.83, respectively (Figure 2). Conversely, Longde county had the lowest number of cases (220) and an annual incidence rate of 9.64/100,000. Notably, the greatest disease burden was from Wuzhong city, encompassing Yanchi county, Tongxi county, and Hongsibao district, which share borders with Inner Mongolia, Gansu, Shaanxi, and Shanxi provinces. Additionally, a spatial autocorrelation analysis based on the annual average incidence revealed a significant spatial cluster at the county level, with a Moran Index of 0.534, a *Z* score of 2.56, and a *p*-value ≤0.05 (0.01). Subsequently, a local autocorrelation analysis indicated a high–high cluster area in Hongsibao district and found five low–low cluster areas, including Pingluo county, Helanshan city, Xiaxi district, Jinfeng district, and Yongning county (Supplementary Figure S1).

### Geographic distribution of species/biovars and host profiles of *Brucella* strains

A total of 230 *Brucella* strains were collected, and isolated between 1950 and 2019 (Table 1), out of which 138 strains were collected from 1950s to 1980s, and identified them based on conventional bio-typing approaches. The remainder 92 strains were isolated from 2009 to 2019, and confirmed as *B. melitensis* through detection of a 731 bp *B. melitensis* specific band, conventional bio-typing tests used to determinate the biovar of 92 *B. melitensis* strains. The 230 strains were collected from 14 regions, with the majority originating from Lingwu City (*n* = 58), followed by 48 from Yanchi, 32 from Wuzhong county, and 24 from Yinchuan. The range of strains in the other 11 areas was 1–24 (Figure 2 and Table 1). The 230 *Brucella* strains in the present study exhibited high species/biovar diversity, encompassing four species and 12 biovars (Table 2). Among these, *B. melitensis* strains were the predominant population in this region. Furthermore, *B. melitensis* strains exhibited the highest host diversity, which included humans, cattle, sheep, and dogs. *B. abortus* was found in cattle and sheep, while *B. suis* and *B. canis* were isolated from humans and dogs (Table 2).

**Table 1:** The regional distribution of *Brucella* strains from 1950 to 2019.

Counties	50s	60s	70s	80s	2009–2013	2019	Total
Xixia	1	5	13	4	1	/	24
Helan	/	9	8	1	/	/	18
Pingluo	/	7	1	4	2	/	14
Dawukou	/	/	4	2	/	/	6
Huinong district	/	5	/	/	/	/	5
Lingwu	/	54	/	/	/	4	58
Zhongning	/	/	/	1	/	/	1
Tongxin	/	/	1	/	/	/	1
Shapotou district	/	/	/	6	/	/	6
Yanchi	/	/	1	1	12	34	48
Pengyang	/	/	/	2	/	/	2
Jingyuan	/	/	/	1	/	/	1
Haiyuan	/	/	/	7	/	/	7
Litong district	/	/	/	/	17	3	20
Qingtongxia district	/	/	/	/	10	2	12
Hongshibao	/	/	/	/	2	/	2
Total	1	80	28	29	44	43 (5^#^)	230

“/”: no strain. “#”: the location of one strain was unknown, and four strains were from border regions, including three in Inner Mongolia, and one in Shaanxi Province.

**Table 2:** Host species, species/biovars, and proportions of isolated *Brucella* strains from 1950 to 2019.

Host	*B. melitensis*			*B. abortus*						*B. suis*		*B. canis*	Total	Proportion (%)
	bv. 1	bv. 2	bv. 3	bv. 1	bv. 3	bv. 4	bv. 5	bv. 6	bv. 7	bv. 1	bv. 3			
Patient	21	8	130	/	/	/	/	/	/	4	2	/	165	71.74%
Cattle	/	1	/	3	7	1	/	3	/	/	/	/	15	6.52%
Sheep	18	18	6	/	/	/	1	1	2	/	1	/	47	20.43%
Dogs	/	1	/	/	/	/	/	/	/	/	/	2	3	1.30%
Total	39	28	136	3	7	1	1	4	2	4	3	2	230	100%

“/”: no strain.

### MLVA genotyping characteristics of 105 *B. melitensis* strains

**Figure 3:**
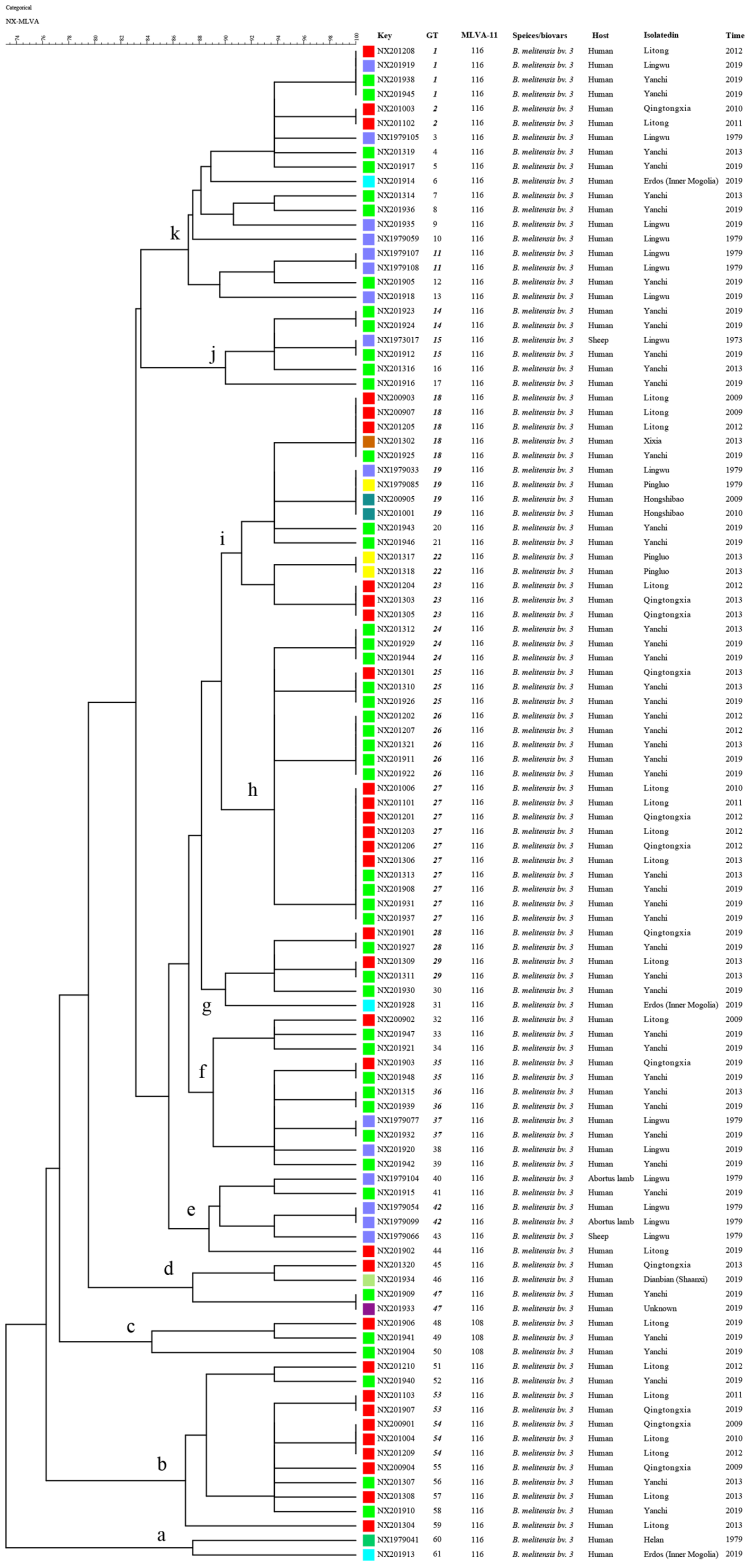
MLVA dendrogram of 105 strains from this study. The columns show the identification numbers (key), MLVA-16 genotypes (GT), and MLVA-11 genotypes (panels 1 and 2A), species/biovar., host species, geographic location, and the year of strain isolation. Regions of isolated strains are indicated by color, and shared MLVA-16 genotypes are indicated by black, bold, italic font.

Based on the *HGDI*, the *MLVA-16* panels exhibited a high discriminatory power for *B. melitensis*, with polymorphism levels of 0.9815 (Table 1). The eight loci of panel 1, and *bruce21* of panel 2A, showed only one allele (*HGDI* = 0). In contrast, the greatest variability was detected in panel 2B (diversity index ≧ 0.6548) within loci *bruce04* (*HGDI* = 0.8190), *bruce16* (*HGDI* = 0.8176), and *bruce30* (*HGDI* = 0.6548). In Panel 1 (*MLVA-8*), all strains exhibited genotype 42, while two *MLVA-11* genotypes were identified: 116 (*n* = 102) and 108 (*n* = 3), with 116 being the dominant circulating genotype and widely distributed throughout the area. This data suggests that all strains belong to the East Mediterranean lineage with a common geographic origin. Furthermore, 105 strains clustered into 11 clusters (*a* ~ *k*) harboring the 61 *MLVA-16* genotypes (*GTs* 1–61) (Figures 2, 3), 39 of which were represented by unique strains. The other 22 were shared genotypes, with associated genotypes shared between two and ten isolates. The 22 shared genotypes comprised 66 clustered strains, with a clustering rate of 62.9% (66/105; Figure 3). These data revealed a co-existing transmission pattern, predominantly of epidemiologically related cases and limited sporadic cases. The most frequently observed genotype, genotype 27, was present in ten isolates obtained from three regions over a decade. The five strains containing the second most frequently observed genotypes, genotypes 18 and 25, were isolated from three regions. Seven shared GTs (GTs 1, 15, 19, 25, 29, 35, and 47) were each found in two to four isolates from different counties. These data suggest that multiple cross-regional transmission events were caused by a common source of infection. Another seven shared GTs (GTs 2, 11, 22, 23, 42, 53, and 54) consisted of strains from the same counties; additionally, strains with defined epidemiological links isolated from two families (*NX201201* and *NX201206*; and *NX201303* and *NX201305*) clustered into identical genotypes (Figure 3), demonstrating that multiple outbreak events within the same county were caused by a common source, respectively. Furthermore, 13 shared GTs containing strains isolated in different years (Figure 3) indicate the ongoing endemic presence of predominant circulating genotypes in this region.

### Investigation of molecular epidemiological relationships at the two scales

**Figure 4:**
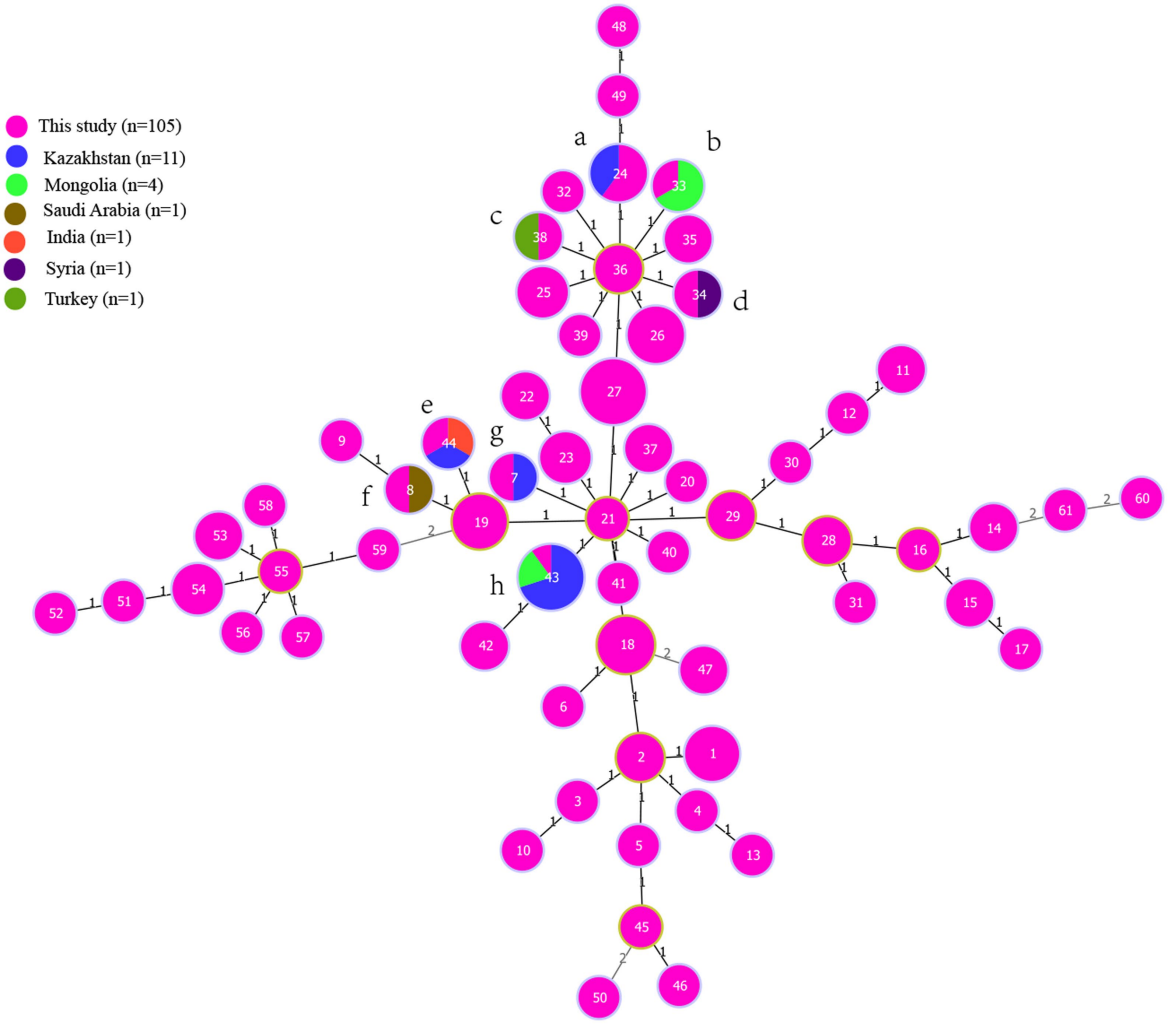
The genetic comparison of strains from the present study to strains from global scale. MST for *Brucella melitensis* generated using the MLVA-16 data from 750 strains from East Mediterranean lineage from 32 countries (Supplementary Figure S2). Finally, 19 strains from seven countries (Supplementary Table S5) that have closely relationship to strains from the present study were selected to further compared the genetic relationship. The MST of strains were constructed by the PHYLOZIV 2.0 software, and color-coding according to country where strains isolated (pink in this study, blue in Kazakhstan, brown in Saudi Arabia, green in Mongolia, purple in Syria, orange in India, blackish green in Turkey), and circle size indicate the numbers of strains. The strains from this study and other countries formed eight **(A–H)** complete match MLVA-16 genotypes, the number on the branch line represents the number of allele differences.

To investigate the molecular epidemiological relationships among strains, an *MLVA-16* comparison analysis was conducted on both national and international scales. A total of 1,330 *B. melitensis* strains from 30 provinces/regions, previously reported in our study, were utilized for this purpose. The *MLVA* comparison revealed that 21 shared genotypes (1–21) existed between strains from Ningxia and those from 31 other provinces (Supplementary Table S4). Strains from these shared genotypes almost spanned all provinces, including Inner Mongolia, Xinjiang, Qinghai, Shanxi, Hebei, Liaoning, and Guangdong, as well as Guangxi, Jiangsu, Jiangxi, Fujian, Zhejiang, and Hainan provinces. Internationally, strains from this study exhibited identical *MLVA-16* genotypes with strains mostly from Kazakhstan (A, E, G, and H), and Mongolia (B,H), but also from Turkey (C), Syria (D), India (E), and Saudi Arabia (F) (Figure 4).

## Discussion

In this study, we performed a preliminary analysis of the epidemiological and clinical characteristics of human brucellosis in Ningxia. We observed three phases of human brucellosis prevalence: the epidemic phase, control phase, and re-emergence phase. The incidence of human brucellosis was high during 1955–1978, then decreased dramatically during 1979–1994, after which brucellosis reemerged in 1995. The observed re-emergence phase is consistent with that reported in Shanxi, in which the incidence increased dramatically from 7.0/100,000 in 2005 to 23.5/100,000 in 2014, with an average annual increase of 14.5% (Chen et al., 2016). However, some regions have not experienced a re-emergence phase. Notably, no positive cases have been reported after 15 years of continuous serological surveillance in Zhongwei county. Consequently, the brucellosis surveillance point in Zhongwei county was discontinued in 2005, resulting in a lack of prevention and control measures in this district. The re-emergence of human brucellosis is associated with many factors. Key contributing factors include the long-term persistence of infection in livestock (sheep and goats) and their movement. Additionally, with the continuous expansion of animal husbandry and the frequent introduction of livestock, resources for quarantine, immunization, and compensation are inadequate, resulting in the long-term persistence of animal brucellosis epidemics. Previous efforts to address this contributing factor have included newborn immunization of female lambs for three consecutive years between 2015 and 2017 to curb the rapid spread (Duan et al., 2023). This resulted in the formation of an effective immunization barrier, and consequently, the incidence of human brucellosis decreased rapidly during this period. Therefore, the implementation of ongoing animal immunization is recommended in Ningxia.

Our results revealed that the incidence of brucellosis was concentrated in Yanchi county, Hongsibao district, and Tongxin county. These data raise questions about the effectiveness of public health measures. This is consistent with a previous study that indicated that high-incidence areas were mostly concentrated in central Ningxia and displayed a certain degree of correlation with the number of sheep in stock (Zhao et al., 2020). These counties are renowned for sheep breeding; for instance, Yanchi County was designated as the National Animal and Poultry Genetic Resources Reserve in 2000. Furthermore, this county also successfully registered the Yanchi Tan Sheep breed as a National Brand within the national germplasm resources core protection area in 2021.

Moreover, Ningxia is home to the largest concentration of Hui people in China, whose diet and lifestyle practices align closely with Islamic traditions. The primary sources of income and animal protein for the local population are sheep and goat farming. While this industry forms the backbone of the county’s poverty reduction efforts, the processing, sale, and consumption of mutton may pose infection risks to humans. This offers a plausible explanation for why the strains from Yanchi county led to multiple-point outbreaks of human brucellosis. A similar effect was observed in Shaanxi, where the increased brucellosis incidence was closely related to the development of large-scale small ruminant (goat and sheep) farms in Guanzhong and some southern Shaanxi regions (An et al., 2021). Notably, Yanchi County is located in the easternmost region of Ningxia, adjacent to the Inner Mongolia Autonomous Region, Gansu (Wei et al., 2019), and Shaanxi (An et al., 2022), which are cluster areas of human brucellosis (Yang et al., 2020). Previous studies showed that the highest seropositivity was observed in Yulin City, Shaanxi, which borders regions that have a high incidence of human brucellosis, including Inner Mongolia, Ningxia, and Gansu (An et al., 2022). The high disease burden in this region raises questions about the effectiveness of public health measures. Therefore, further genome molecular association surveys of strains from the border regions are necessary to elucidate the transmission chain.

In this study, we observed high species/biovars and host diversity in Ningxia, constituting a relatively complete niche for the spread of *Brucella* strains. The potential for cross-host transmission of *Brucella* strains may create an optimal environment for their persistent circulation in this region. The results from MLVA genotyping suggested that the dominant circulating genotypes persistently expanded over an extended time span, contributing to both intra-county and cross-county human brucellosis epidemics. Moreover, the 39 singletons each represented one strain, indicating sporadic cases, including four strains from the border regions of Inner Mongolia and Shaanxi Province. This is consistent with the results of MLVA-16 genotyping of 70 *B. melitensis* isolates from Egypt, which revealed that 51 genotypes were represented by single isolates, while the remaining 19 genotypes were shared among 67 isolates, suggesting a combination of sporadic and epidemiologically related characteristics in *B. melitensis* infection (Sayour et al., 2020). Further genomic epidemiological studies are necessary to support the present results and illustrate the phylogenetic relationships of *B. melitensis* strains. *B. melitensis* has been the dominant circulating population for nearly eight decades, responsible for the majority of human brucellosis cases. A similar high prevalence of *B. melitensis* has been observed in small ruminants in Turkey, as evidenced by isolations from sheep, goat, and cattle abortion samples from various farms across seven geographical regions (Akar and Erganis, 2022). Furthermore, *Brucella* strains isolated from all regions of Kyrgyzstan were identified as *B. melitensis* (Kydyshov et al., 2022). Notably, Guangxi province has witnessed considerable shifts in the species/biovar composition of *Brucella* strains, with previously predominant populations, *B. suis* and *B. canis*, being replaced by recently emerging *B. melitensis* strains (Liu et al., 2019). Furthermore, the predominant circulating genotypes in this region were similar to strains from the Northwest, including Inner Mongolia, Xinjiang, and Qinghai. These data indicate that brucellosis has become a significant regional issue, with a high risk of spillover to southern areas due to animal trade and their products. Additionally, strains from this study showed complete consistency in *MLVA-16* genotypes with strains from other Asian countries, such as Kazakhstan and Mongolia. The breeding and trade of small ruminants have contributed to the strain’s spread due to similar lifestyle and domestic farming practices in these agricultural and pastoral areas in China, Kazakhstan, and Mongolia. WGS-SNP analysis showed that strains from these countries lack clear territorial differentiation, demonstrating that strains continuously expand and spread in countries along the Silk Road (Liu et al., 2019). The active exchange and trade of sheep and goats among these countries are one of the main driving factors. Further genomic epidemiology investigation of strains from multiple hosts will provide a new genetic map to better depict the transmission route and evolution profile of strains.

Based on the interpretations of our results, it is recommended to strengthen publicity and education and mobilize farmers and herders to consciously participate in brucellosis control work. First, enhancing communication and education collaboration with agricultural and animal husbandry departments to raise awareness should be a focus, particularly among high-risk groups. This should include promoting healthy lifestyles to reduce the epidemic of brucellosis (Dadar et al., 2020; Gong et al., 2023). Second, high-risk populations should be educated about the risks associated with privately slaughtering, trading, transferring, or consuming animals infected with brucellosis, those that died of disease, or those that died of unknown causes, especially during the spring calving and lambing season. They should be encouraged to implement rigorous personal protection and field disinfection measures (Abu Shaqra, 2000). Additionally, the migration of sheep from high-to low-infection areas should be strictly limited to prevent further spread (Zhang et al., 2014). Moreover, targeted prevention and control measures should be implemented, including collaborative publicity campaigns with local Islamic church organizations, to ensure that local people can participate in monitoring and control while respecting different cultural differences.

Although there were some new insights in this study, there may be potential limitations to be considered. Firstly, whole genome sequencing of some representative strains is needed to better illustrate the epidemiological transmission pattern and evolution characteristics of strains. Secondly, the limited number of strains from animals suggests that future studies should include larger sample sizes to verify the results of this study.

## Conclusion

Our analysis indicates an ongoing increase in the incidence rate of human brucellosis in Ningxia, from 0.441 (per 100,000) in 2005 to 86.83 (per 100,000) in 2022. Geographically, the affected area has continuously expanded, progressing from one affected county in 2004 to all 22 counties by 2022. *B. melitensis* emerged as the dominant circulating strain, responsible for the majority of cases. Molecular epidemiological investigations conducted using MLVA-16 genotyping have revealed the persistence of dominant genotypes in both intra-county and cross-county epidemics of human brucellosis. Brucellosis has become a serious regional issue, and there is a high risk of spillover to southern areas due to the trade in animals and their products.

The results of this study have provided a foundation for recommendations on the prevention and control of brucellosis epidemics. Further genomic epidemiology studies of circulating *Brucella* strains will provide new insights into the evolving epidemiological patterns of human brucellosis in Ningxia.

## Data availability statement

The original contributions presented in the study are included in the article/Supplementary material, further inquiries can be directed to the corresponding authors.

## Ethics statement

The studies involving humans were approved by Ethics Committee of the Ningxia Fourth People’s Hospital (no. NXFPH-0624). The studies were conducted in accordance with the local legislation and institutional requirements. Written informed consent for participation in this study was provided by the participants’ legal guardians/next of kin.

## Author contributions

GL: Methodology, Funding acquisition, Investigation, Writing – review & editing. XM: Investigation, Methodology, Writing – review & editing, Resources. RZ: Writing – review & editing, Formal analysis, Project administration. JL: Supervision, Writing – review & editing. PZ: Writing – review & editing, Data curation, Methodology. BL: Project administration, Writing – review & editing, Resources, Supervision. TL: Data curation, Writing – review & editing, Software. HR: Supervision, Writing – review & editing, Data curation, Formal analysis. ZgL: Data curation, Software, Conceptualization, Methodology, Writing – original draft. ZjL: Supervision, Writing – review & editing, Formal analysis, Project administration. XJ: Project administration, Supervision, Writing – review & editing.
