# Supplementary figures and images for: Epidemiological changes and molecular characteristics of Brucella strains in Ningxia, China
Source: Front Microbiol. 2024 Jan 19;15:1320845. doi: 10.3389/fmicb.2024.1320845 (PMC10835715; doi:10.3389/fmicb.2024.1320845)

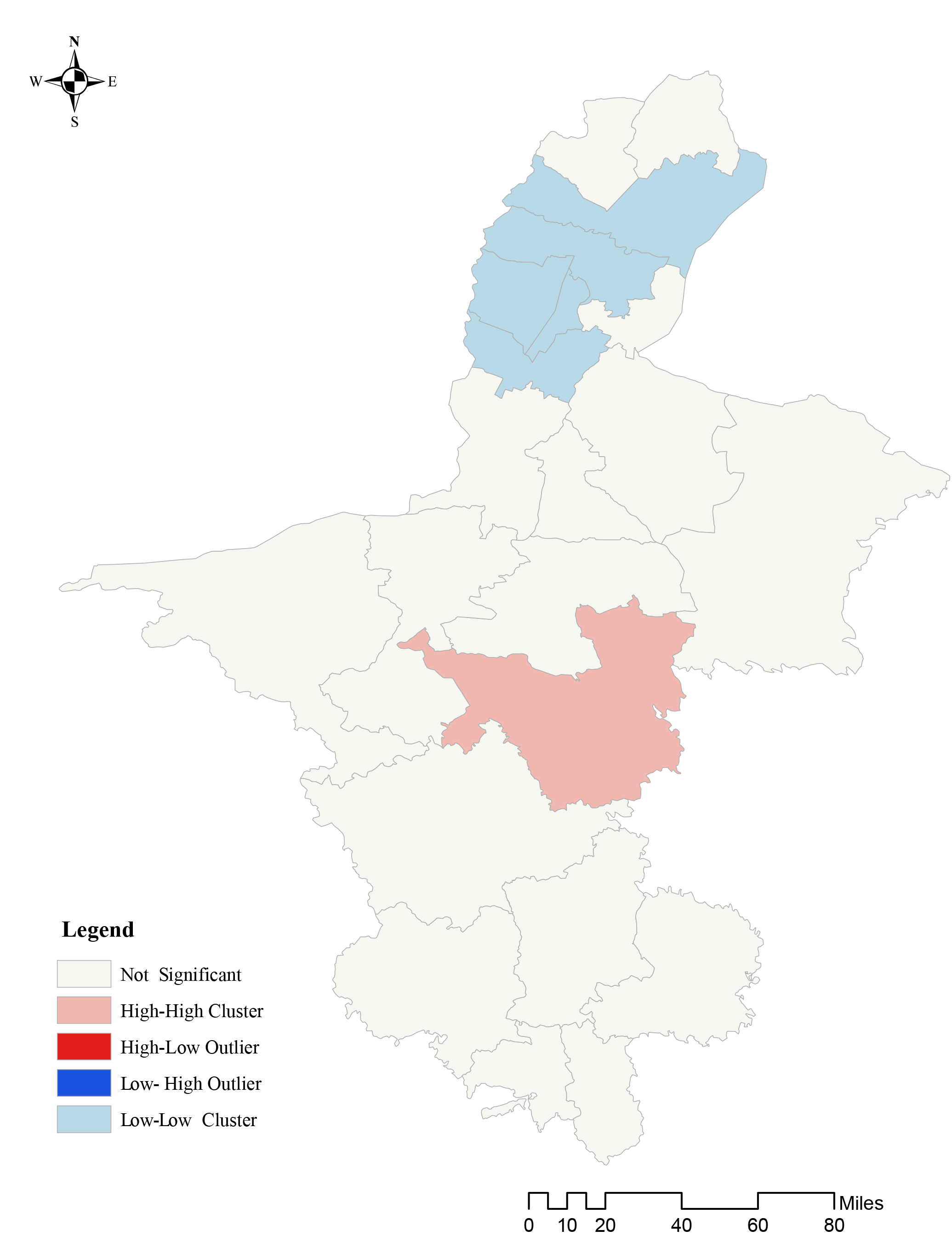

Supplement: Supplementary file 5 [file Image_1.TIF]

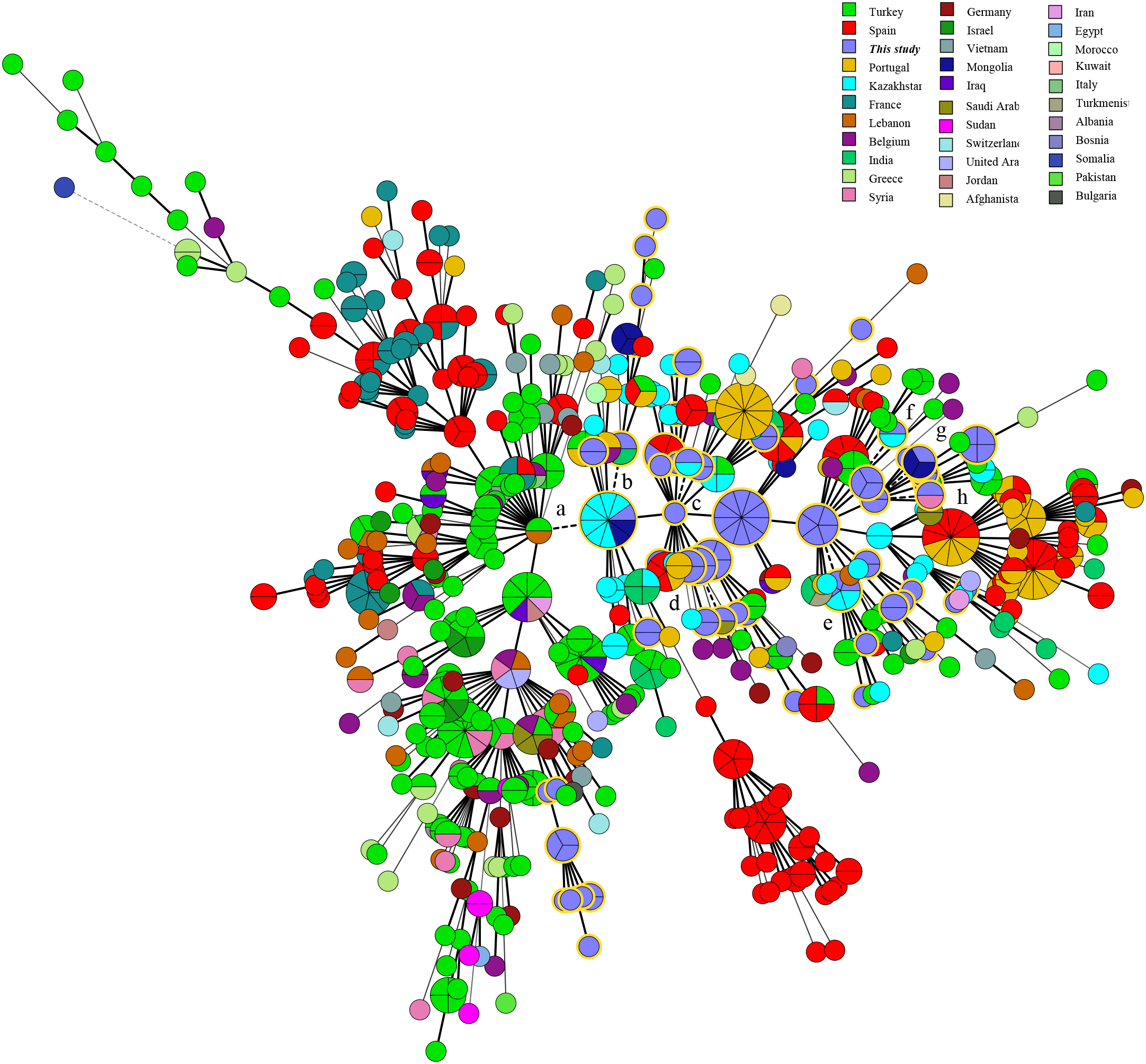

Supplement: Supplementary file 6 [file Image_2.TIF]
